# Effect of Remote Ischaemic Preconditioning on Liver Injury in Patients Undergoing Major Hepatectomy for Colorectal Liver Metastasis: A Pilot Randomised Controlled Feasibility Trial

**DOI:** 10.1007/s00268-016-3823-4

**Published:** 2016-12-08

**Authors:** Sanjeev Kanoria, Francis P. Robertson, Naimish N. Mehta, Giuseppe Fusai, Dinesh Sharma, Brian R. Davidson

**Affiliations:** 1 Hepato-Pancreatico-Biliary and Liver Transplant Unit, University Department of Surgery, Royal Free Hospital, London, NW3 2QG UK; 2 Department of Surgical and Interventional Science, Royal Free Campus, University College London, 9th Floor Royal Free Hospital, Pond Street, London, NW3 2QG UK

## Abstract

**Background:**

Liver resection produces excellent long-term survival for patients with colorectal liver metastases but is associated with significant morbidity and mortality from ischaemia reperfusion injury (IRI). Remote ischaemic preconditioning (RIPC) can reduce the effect of IRI. This pilot randomised controlled trial evaluated RIPC in patients undergoing major hepatectomy at the Royal Free Hospital, London.

**Methods:**

Sixteen patients were randomised to RIPC or sham control. RIPC was induced through three 10-min cycles of alternate ischaemia and reperfusion to the leg. At baseline and immediately post-resection, transaminases and indocyanine green (ICG) clearance were measured.

**Findings:**

The RIPC group had lower ALT and AST levels immediately post-resection (ALT: 43% lower 497 ± 165 vs 889 ± 170 IU/L; *p* = 0.019 AST: 54% lower 408 ± 166 vs 836 ± 167 IU/L; *p* = 0.001) and at 24 h (ALT: 41% lower 412 ± 144 vs 698 ± 137 IU/L; *p* = 0.026 AST: 50% lower 316 ± 116 vs 668 ± 115 IU/L; *p* = 0.02). ICG clearance was reduced in controls versus RIPC immediately after resection (ICG-PDR: 11.1 ± 1.1 vs 16.5 ± 1.4%/min; *p* = 0.035).

**Conclusions:**

This pilot study shows that RIPC has potential to reduce liver injury following hepatectomy justifying a prospective RCT powered to demonstrate clinical benefits.

## Introduction

Liver resection for colorectal metastasis is the gold standard treatment and has improved survival in patients with colorectal liver metastasis [1, 2]. Warm ischaemia reperfusion injury (IRI) to the liver occurs during major liver resections with mobilisation and retraction of the liver and with the use of temporary portal inflow occlusion (Pringle manoeuvre) [3]. Even in the absence of inflow occlusion, oxygenation of the liver tissue is significantly reduced during mobilisation prior to parenchymal transection [4], resulting in repetitive warm IR injury and significant hepatocyte death prior to parenchymal transection and inflow occlusion [5, 6]. Livers with fibrosis, steatosis or following neo-adjuvant chemotherapy [7] are more susceptible to this warm IR injury. The mean age of patients undergoing liver resection is 60 years [8], and more patients are undergoing neo-adjuvant chemotherapy. As such, strategies to ameliorate IRI are a key clinical concern especially in this group of patients.

Various strategies to reduce IRI to the liver have been described including ischaemic preconditioning (IPC), which may be applied directly or remotely. Ischaemic preconditioning reduces the adverse effects of IRI through previous exposure to a brief period(s) of vascular occlusion. Direct IPC has been shown to reduce IRI in experimental models of warm hepatic IRI [9] and in patients undergoing major liver resections [10, 11]. However, experimental studies have shown that direct IPC through clamping of the portal inflow vessels may impair liver regeneration [12, 13] and in a multivariate analysis of patients undergoing liver resection, this was found to be an independent predictor for increased post-operative morbidity [14].

Novel methods of preconditioning without direct stress to the liver include remote ischaemic preconditioning (RIPC) [15]. In RIPC, a remote organ is preconditioned prior to ischaemia of the target organ, and in experimental studies, this has been shown to reduce IRI to the myocardium [16], the liver [17, 18] and other organs [15]. The beneficial effect of RIPC in reducing IRI was first demonstrated in paediatric patients undergoing cardiopulmonary bypass [19]. Results from following clinical trials and meta-analyses in cardiac and vascular surgery have been varied [16, 20–26]. It is unclear why some trials have shown evidence of protection while others have not. This may reflect underlying comorbidities, different conditioning protocols or variability in the potential for a target organ to be preconditioned. As the mechanism by which RIPC provides protection remains unknown, it is difficult to understand what is the reason for these differing results.

We have previously shown that RIPC can reduce liver IRI in an experimental model [17]. This current trial is a proof of concept study to determine whether RIPC reduces IRI in patients undergoing major liver resectional surgery and to help determine potential end points for a subsequent trial to determine the risks and benefits of RIPC in patients undergoing major liver resection for colorectal liver metastases.

In this study, in addition to measuring biochemical parameters of hepatocellular injury, indocyanine green (ICG) clearance from the liver and ICG plasma disappearance rate (ICG-PDR) has been measured which provide reliable early indicators of post-operative liver function [27]. To predict liver injury after hepatectomy and to assess functional hepatic reserve, static measures of liver function such as transaminases [28] have limited reliability. Dynamic tests using ICG clearance and lidocaine metabolism are superior with ICG having the advantage of being measured non-invasively [29]. There is a close correlation between the ICG-PDR and ICG-retention rates measured non-invasively and their corresponding values calculated by conventional ICG methods [30].

### Hypothesis

The hypothesis was that RIPC would be safe and feasible in patients undergoing liver resection surgery and that RIPC would result in evidence of a reduction in peri-operative liver injury.

## Materials and methods

A single-centre blind prospective randomised controlled trial was performed at the Royal Free Hospital, between April 2005 and April 2007, following approval by the local NHS ethical board (54,561,358). The trial involved randomisation of patients undergoing major liver resection (3 segments or more) for colorectal liver metastasis and was carried out in conjunction with a similar trial in liver transplant recipients. It was registered with ClinicalTrials.gov: Number NCT00796588.

Patients above the age of 18 being considered for major liver resection for colorectal liver metastasis under 3 surgeons were enrolled in the study. Exclusion criteria included: the absence of written informed consent, peripheral vascular disease, blood disorders, e.g. sickle cell disease, localised limb infections, pregnancy, severe co-morbid disease, uncontrolled diabetes and sepsis.

Twenty-two patients were assessed for eligibility following which 6 were excluded and 16 were randomised into a control and a RIPC group. For randomisation, computer-generated random numbers were generated and stored in sealed envelopes which were opened following induction of anaesthesia. Patients were blinded to the intervention (RIPC or sham), but the surgeon was not.

### The preconditioning stimulus

**Fig. 1:**
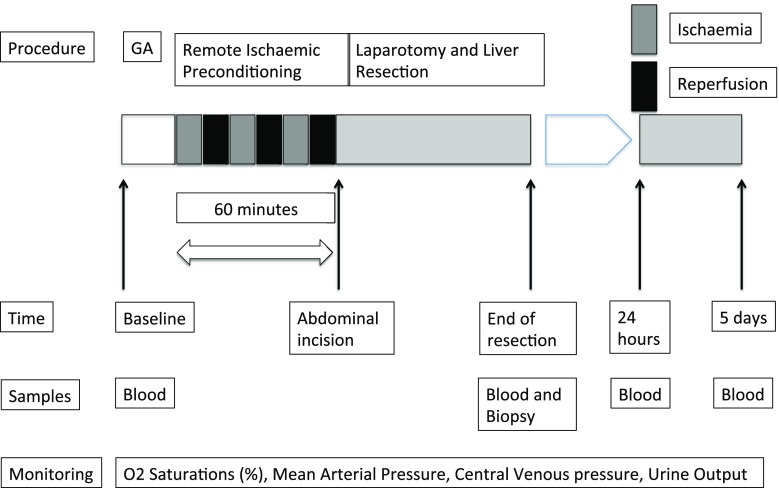
Trial protocol

In the control group, a sham consisted of a pneumatic tourniquet being placed on the right upper thigh without being inflated. In the RIPC group, following general anaesthesia but before the abdominal incision, the lower limb was covered with two layers of stockinette and elevated to 45° for 3 min. A wide pneumatic tourniquet was applied to the right upper thigh in accordance with safe and recommended practices by the Association of Peri-operative Registered Nurses (AORN) [31]. To induce RIPC, the tourniquet was inflated to twice the measured systolic arterial pressure for 10 min and then deflated for 10 min to reperfuse the leg. This was repeated twice and completed prior to commencing the operation (Fig. 1).

### The surgical procedure

Liver resection was performed through a hockey stick incision. Inflow vessels on the side of the resection were divided extra-parenchymally. Portal vascular inflow occlusion (Pringle manoeuvre) was not required in any of the patients in the study. Liver transection was performed using an ultrasonic dissector in all cases. All patients received antibiotic prophylaxis and post-operatively a daily subcutaneous injection of low molecular weight heparin as thrombo-prophylaxis.

## Data collection and measurements

### Complications

During the preconditioning stimulus, patient’s haemodynamics were continually monitored. Patients were clinically examined post-operatively for evidence of deep vein thrombosis (DVT) or pulmonary embolus (PE) formation and limb paraesthesia or pain. Doppler ultrasound or CT pulmonary angiogram was requested if there was clinical suspicion of embolus formation.

### Blood measurements

In both groups, 35 mls of peripheral blood was collected at the following time intervals: baseline (following induction of anaesthesia), at the end of the liver resection, and 24 h post-operatively. Whole blood samples were collected from the arterial line in precooled tubes for the measurement of serum transaminases, serum bilirubin, urea and electrolytes. Measurements were made using an automated clinical chemistry analyser (Hitachi 747, Roche Diagnostics Ltd., Sussex, UK).

### Indocyanine green (ICG) pulse densitometry

ICG is a fluorescent dye eliminated exclusively by the liver, and its elimination rate is used to evaluate global liver function. A bolus of 50 mg ICG (dry powder) [Limon, Pulsion, Munich, Germany] was dissolved in the supplied solvent giving a concentration of 5 mg dye/ml solvent. This was injected slowly intravenously in a dose of 0.5 mg/kg through the central line. Both absorption and emission spectrum of ICG are in the near-infrared range, and its concentration can be quantified by actual absorbance either invasively with a fibre-optic catheter or non-invasively through the skin [32]. In this trial, the blood concentration of ICG was measured non-invasively via an optical probe attached to the patient’s finger and connected to a trans-cutaneous pulse densitometry monitor (Limon, Pulsion Medical Systems AG, Munich, Germany). Measurements were made at baseline and immediately following completion of liver resection and were recorded as plasma disappearance rate of ICG [ICG-PDR (%/min)] and ICG retention rate after 15 min (*R* 15%).

### Histological examination

The resected liver specimen was fixed in 10% formalin. Tissues were embedded in paraffin and stained with haematoxylin–eosin for histological examination. According to the Royal College of Pathologists guidelines, the resected specimen was evaluated for resection margins, nature, distribution and differentiation of the tumour. The normal liver parenchyma was examined for significant signs of IRI, steatosis and fibrosis. Histological evidence of IRI included portal tract inflammation with neutrophil infiltration, hepatocyte ballooning or apoptosis/necrosis and disruption of the trabecular architecture around the central lobar vein. The reporting pathologist was blinded to the trial arm that the patient was allocated to.

### Statistical analysis and power calculations

Although power calculations are not deemed necessary for a pilot feasibility trial, previous studies in direct IPC have achieved a 50% reduction in serum transaminases in comparison with the control group at 24 h post-reperfusion [10, 33]. To demonstrate a benefit of RIPC in reducing liver injury as indicated by a reduction in serum transaminases with a statistical significance (*p* < 0.05), a power of 80% (two-tailed test of proportions), an *α*-error of 0.05 and a *β*-error of 0.00, it was calculated that a sample size of at least 8 patients per group was required. Distribution of data was analysed by Shapiro–Wilk test and Q–Q plots. Continuous data were expressed as mean (±SD), and comparisons between groups were tested by unpaired Student’s *t* test as appropriate. Dichotomous data were presented as a proportion of the whole and comparisons between groups were tested by Chi-squared tests. A *p* value of <0.05 was considered significant, and analysis was by intention to treat.

## Results

**Fig. 2:**
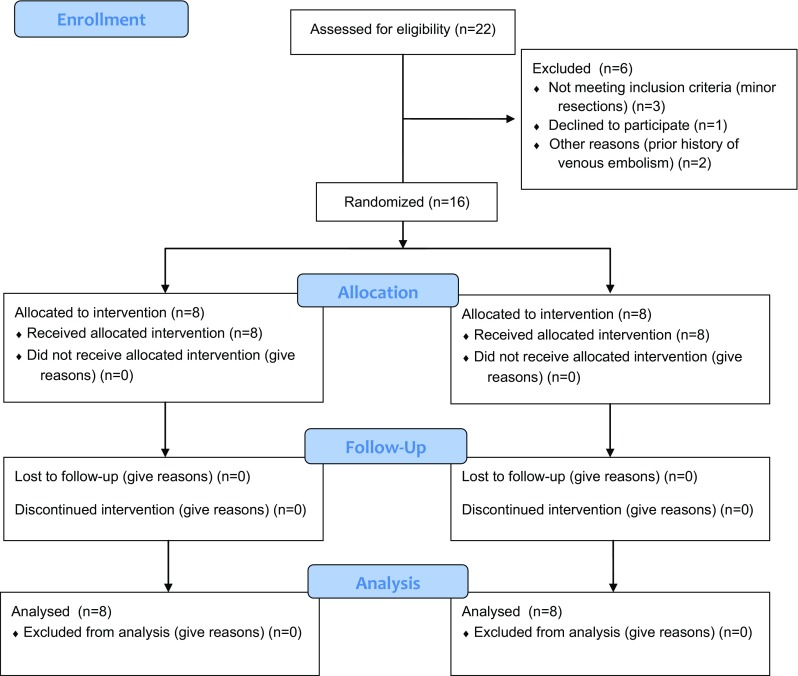
Randomisation according to the CONSORT guidelines

Twenty-two patients undergoing liver resection surgery for colorectal liver metastases were approached with 1 patient not wishing to participate in the trial. Five patients were further excluded. Two patients were found to have minor peripheral vascular disease. Three patients who were expected to undergo a major resection underwent a multiple wedge resections and were excluded prior to randomisation. Of the remaining 16 patients, 8 were randomised to the control group and 8 were randomised to the intervention (RIPC) group (Fig. 2). Both groups were well matched at baseline including incidence of pre-operative chemotherapy and degree of steatosis (Table 1).

**Table 1:** Demographic data

	Control group	RIPC group
Age (years)	66–74	58–77
Sex ratio (M:F)	6:2	7:1
BMI (kg/m^2^)	17–34	22–34
Operative procedure		
Right hepatectomy	6	5
Extended right hepatectomy	2	2
Left hepatectomy	0	1
Neo-adjuvant chemotherapy	3	2
Duration of operation (h)	6.25 (4.5–8)	6.15 (4.7–7.6)
Intra-operative parameters		
Central venous pressure (mmHg)	10 (8–12)	9 (7–14)
Mean arterial blood pressure (mmHg)	69 (60–77)	70 (65–80)
Blood transfusion (mls)	300 (0–500)	350 (0–750)

### Safety and feasibility

Seventy-three percentage of patients approached were recruited to the trial. All patients randomised to the RIPC group successfully underwent RIPC, and there was a 0% drop out rate post-randomisation.

No patients experienced haemodynamic instability during cuff inflation.

On clinical examination, there was no evidence of DVT or PE formation. No patient required a Doppler USS or CTPA. No patient complained of pain or paraesthesia post-operatively.

### Clinical outcomes

There were no deaths in either group, and no patient suffered from post-operative liver failure. There was a higher incidence of both wound infections (2 vs 1, *p* = 0.38) and basal atelectasis (4 vs 3, *p* = 0.62) in the control group although neither of these were significant. One patient in the RIPC group developed a post-operative pneumonia, and one patient in the control group developed a post-operative intra-abdominal collection that required radiological drainage. Patients who underwent RIPC spent on average longer in ITU post-operatively although this was not significant (2 vs 1.5 days, *p* = 0.46).

### Serum transaminases

**Fig. 3:**
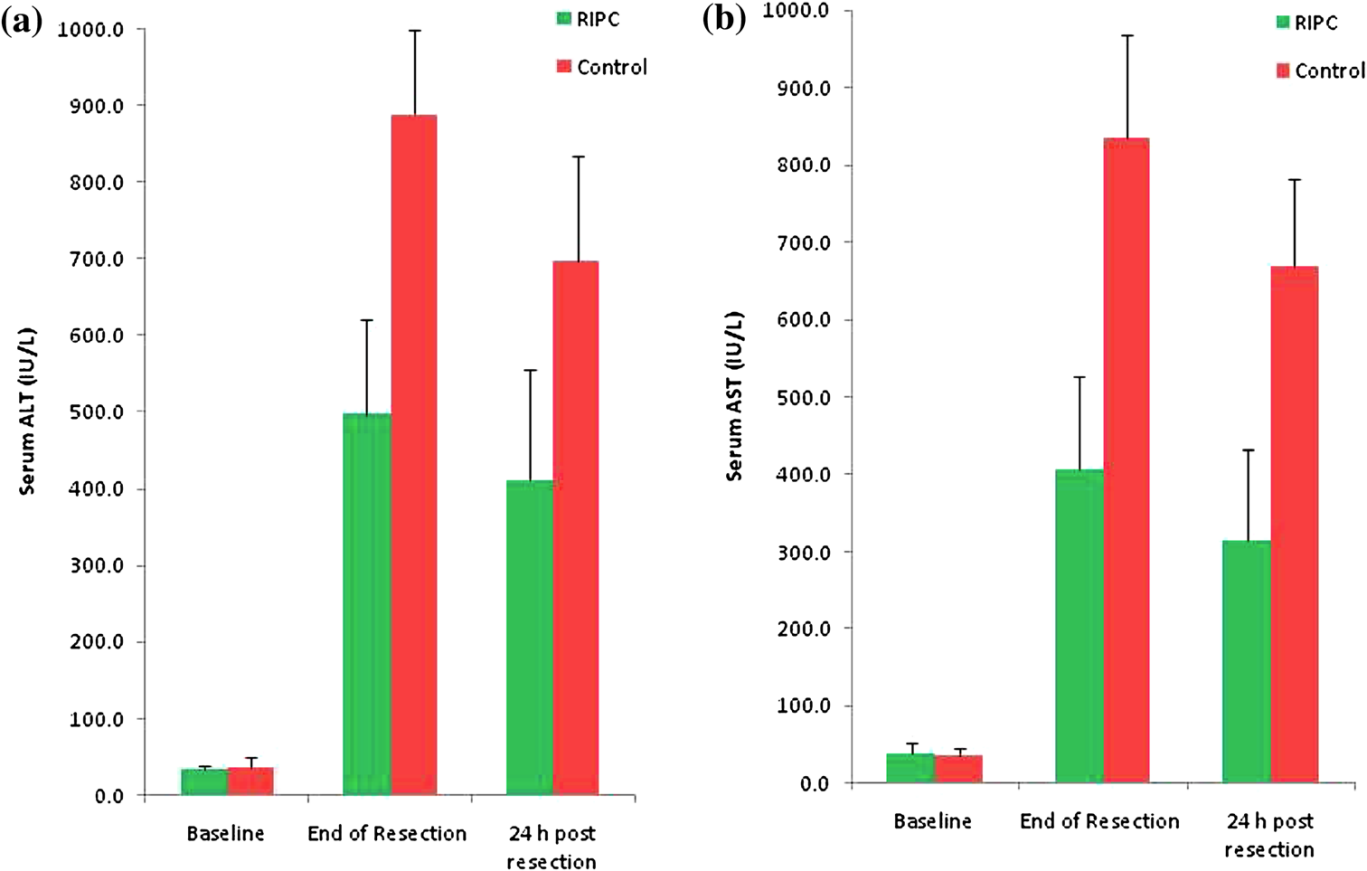
**a** In both groups, serum ALT levels at the end of resection and 24 h post-resection were raised versus baseline. At the end of resection, ALT levels were 43% lower in the RIPC group versus control (*p* = 0.019) and at 24 h was 41% lower versus control (*p* = 0.026). **b** In both groups, serum AST levels at the end of resection and at 24 h post-resection were raised versus baseline. At the end of resection, serum AST levels were 54% lower in the RIPC group versus control (*p* = 0.001). At 24 h post-resection, serum AST levels were 50% lower in the RIPC group versus control (*p* = 0.02)

In both groups, serum ALT levels at the end of the resection and at 24 h post-resection were significantly raised vs baseline (Fig. 3a). At the end of resection, serum ALT levels were 43% lower in the RIPC group compared to the control group (497 ± 165 vs 889 ± 170 IU/L; *p* = 0.019). At 24 h post-resection, ALT levels were 41% lower in the RIPC group than in the control group (412 ± 144 vs 698 ± 137 IU/L; *p* = 0.026).

The pattern was similar for the changes in AST. At the end of resection, serum AST levels were 54% lower in the RIPC group compared with the control group (408 ± 166 vs 836 ± 167 IU/L; *p* = 0.001). At 24 h post-resection, serum AST levels were 50% lower in the RIPC group compared to the control group (316 ± 116 vs 668 ± 115 IU/L; *p* = 0.02) (Fig. 3b). There were no significant differences in mean serum bilirubin levels between the two groups at the measured time points.

### ICG measurements

**Fig. 4:**
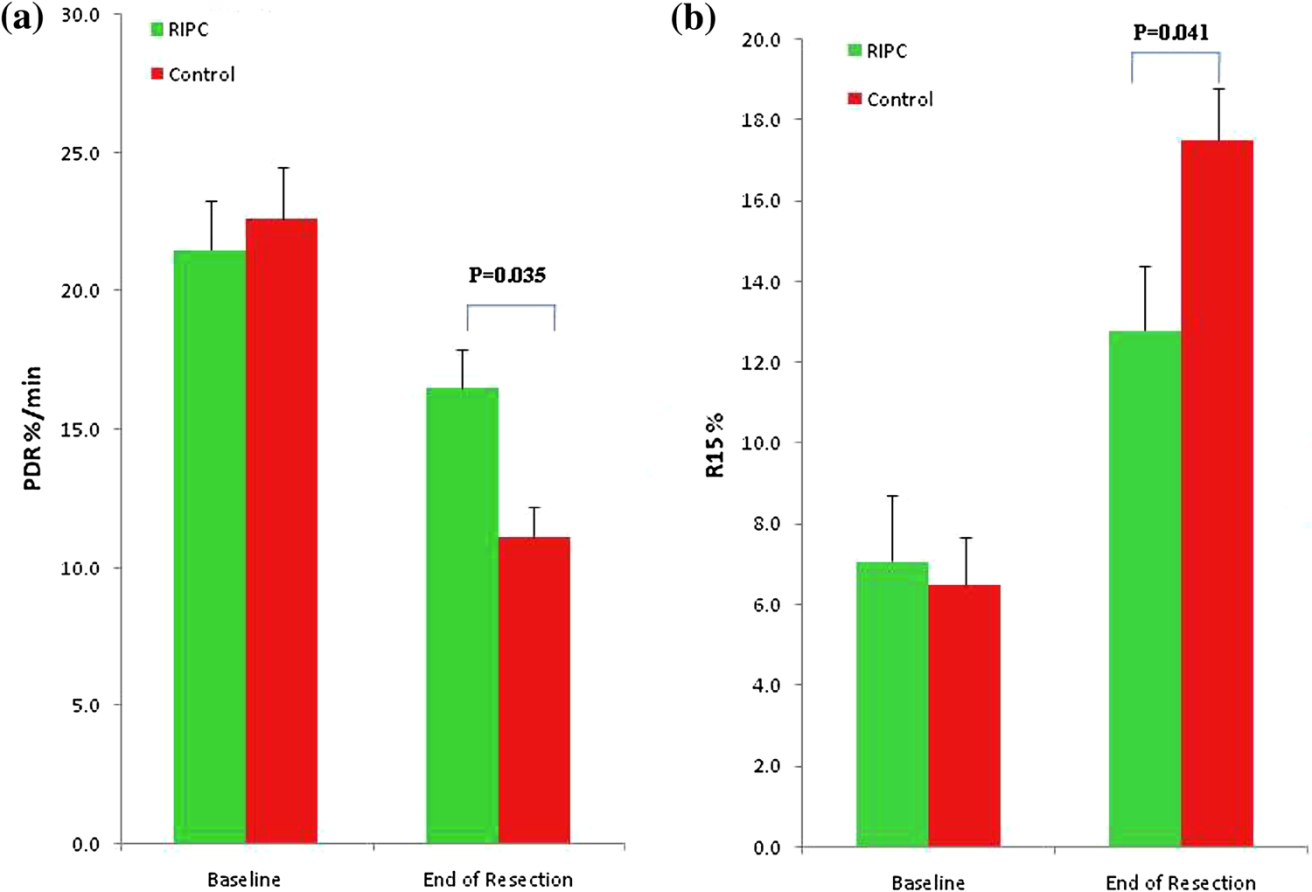
**a** ICG-PDR of <15%/min indicates borderline liver function. ICG-PDR at baseline in the two groups was not different, but immediately after liver resection there was a significant difference between the two groups (*p* = 0.035). **b** ICG retention of >15% after 15 min [ICG-R 15 (%)] after ICG injection is an indicator of severe liver dysfunction. ICG-R 15 (%) at baseline in the two groups were the same, but immediately after liver resection there was a significant difference between the two groups (*p* = 0.041)

The plasma disappearance rate of ICG (ICG-PDR) at baseline in the two groups was similar (control 22.6 ± 1.9 vs RIPC 21.5 ± 1.8%/min). After liver resection, there was a significantly higher ICG clearance in the RIPC group (control 11.1 ± 1.1 vs RIPC 16.5 ± 1.4%/min; *p* = 0.035) (Fig. 4a). Similarly the ICG retention at 15 min [*R* 15 (%)] at baseline in the two groups was similar (control 6.5 ± 1.2 vs RIPC 7.1 ± 1.6%), whereas after liver resection there was a significantly reduced retention of ICG in the RIPC group (control 17.5 ± 1.3 vs RIPC 12.8 ± 1.6%; *p* = 0.041) (Fig. 4b).

### Histological findings

In both groups, histology of the liver from the resected specimens was similar to minimal steatosis. There was no pathological evidence of IRI seen.

## Discussion

This pilot feasibility study has shown that RIPC is safe and feasible in patients undergoing liver resection for colorectal liver metastases. Although the trial was not powered to demonstrate improved clinical outcome, a reduction in liver injury as determined by a significant reduction in post-operative serum transaminases and significantly increased ICG clearance was demonstrated in patients undergoing RIPC prior to liver resection.

Liver resection is regarded as the gold standard of treatment for resectable colorectal metastases [1]. The success of the surgery has led to patients requiring extensive parenchymal resections being offered hepatic resection with the risk of post-operative hepatic insufficiency secondary to insufficient residual liver volume. Following surgical resection morbidity can be up to 36%. Besides the extent of the resection, the finding at surgery of liver steatosis and fibrosis is additional important risk factors for post-operative hepatic insufficiency [34] and impaired liver regeneration [35] due to the increased susceptibility to warm IRI [36, 37]. Patients with colorectal metastasis are usually elder (median age 60 years). In experimental models, aged livers have a greater susceptibility to minor degrees of warm IRI [38].

This study has shown that RIPC reduces markers of hepatocellular injury following liver mobilisation in patients undergoing major liver resections and increases the ICG clearance, an important indicator of liver viability [27]. Studies investigating the release of markers of liver parenchymal damage (transaminases and glutathione S-transferase) at different stages during and after major liver resection have shown that a significant reduction in hepatic oxygenation occurs during mobilisation of the liver [4] and that markers of parenchymal injury and hepatocyte cell death are significantly raised following mobilisation in the absence of inflow occlusion [5, 6]. This is in keeping with the results from this study which has demonstrated a significant increase in serum transaminase levels in the absence of inflow occlusion and demonstrates that RIPC can protect the liver from this parenchymal injury. This is important as the potential benefits RIPC can be provided more globally rather than only in patients undergoing inflow occlusion.

No patients in this trial had steatosis or fibrosis of the liver; however, 5 patients had undergone neo-adjuvant chemotherapy. If these results can be reproduced in a more extensive trial including patients with risk factors for post-operative liver insufficiency (elderly, prolonged chemotherapy, steatosis, reduced residual liver volume), this may translate into a reduction in the morbidity and mortality associated with liver resection.

The two groups in the trial were well matched for baseline clinical characteristics and indications for hepatectomy. Following major liver resection, serum transaminases increase, peak between 24 and 36 h and return to normal levels within 3–5 days [39]. The severity of IRI is reflected by a rise in serum transaminase levels. In this study in all patients in the two groups, both serum AST and ALT levels increased at the end of the liver resection, peaked at 24 h and returned to normal in 5 days. RIPC produced a significant reduction in serum transaminases at the end of surgery and at 24 h post-reperfusion. There was no difference between RIPC and sham groups at 5 days. Direct IPC of the liver in patients undergoing liver resection [10] and liver transplantation [33] also results in a reduction in serum transaminases. However, in experimental studies direct IPC has been shown to impair liver regeneration [12, 13] and is associated with a higher risk of post-operative morbidity in patients undergoing major hepatectomy [14]. Hence, in steatotic and small remnant livers even this short duration of direct stress to the liver may be detrimental. This stress can be avoided through remote preconditioning. A limitation of this trial, however, is that RIPC has not been compared against direct IPC of the liver; however, in such a small pilot trial there was insufficient numbers to perform this. Furthermore, the primary aim of this study was to check for feasibility and safety of limb RIPC. Future large RCTs should incorporate a direct IPC arm to allow for a comparison between IPC and RIPC.

Indocyanine green (ICG) is eliminated by the liver largely unchanged into bile and does not undergo entero-hepatic recirculation. Elimination is dependent on several factors including hepatic blood flow, hepatocellular uptake and biliary excretion. Hence, the rate of disappearance from the plasma [ICG-PDR (%/min)] and its percentage retention in the liver at 15 min [*R* 15 (%)] is a dynamic measure of liver function describing the functional status of the liver at the time of assessment [27]. ICG retention of >15% after 15 min of a bolus injection of ICG is an indicator of significant liver dysfunction and a predictor of reduced patient survival following major liver resections [40]. Measurement of ICG-PDR is more sensitive than serum enzyme tests for assessing liver dysfunction and predicting outcome [41]. In liver transplantation, ICG-PDR measured immediately after liver reperfusion is useful for early diagnosis of primary graft dysfunction and allografts with ICG-PDR of <15%/min have borderline function [42]. Levels below 5%/min are associated with a high risk of graft failure [27]. In this trial, RIPC improved liver function as demonstrated by an increased ICG plasma clearance and a reduced ICG retention when compared with the control group. Although a reduction in liver injury and improved liver function following RIPC has been identified in this study by reduced transaminases and increased ICG clearance, further sensitive markers of liver injury could be incorporated into future trials including liver fatty acid binding proteins and glutathione S-transferase to further clarify the protection gained by RIPC.

A limitation of this study is that it has not investigated for potential mechanisms of the protection of RIPC. Liver injury propagates an inflammatory response [5], and it has been shown in animal models that mice lacking CD4+ T cells are protected from warm hepatic IRI [43]. Analysis of serum cytokines (IL-6, IFNγ and TNFα) during and post-liver resection could be incorporated into future studies to measure the inflammatory response and identify the effect of RIPC on this mechanism of injury.

This study has demonstrated the safety of inducing RIPC using a pneumatic limb tourniquet. A standard orthopaedic tourniquet was utilised in the trial as would be used to perform limb surgery in a bloodless field. Tourniquet-associated complications including pain, paraesthesia, pressure changes and haemodynamic disturbances usually occur continuous inflation for more than half an hour [44] and were not observed at any stage during this trial. Standard safety and utilisation guidelines would appear to be adequate to guide the use of a pneumatic tourniquet for producing remote preconditioning.

The benefit of RIPC has not been previously demonstrated in liver resection surgery. Clinical trials in cardiovascular surgery have shown that RIPC may reduce myocardial injury. The benefit of RIPC was first shown in children undergoing cardiac surgery [19]. Subsequently, RIPC was shown to reduce myocardial injury in patients undergoing coronary artery bypass surgery [16, 22] and the incidence of peri-operative infarcts in those undergoing aortic surgery [21]. Two large recent trials, however, have failed to demonstrated clinical benefit following RIPC in the setting of cardiac surgery [25, 26]. What is clear from these trials is that differing protocols for the preconditioning stimulus have been used with varying numbers of cycles (2 vs 4) and different methods of vascular occlusion (direct vascular clamping vs limb tourniquet). It has previously been shown in animal models that clamping of the femoral pedicle was as efficacious as a limb tourniquet in ameliorating IR injury [45]. What has not been elucidated is the number and length of cycles that are required to effectively precondition humans. In trials of IPC of donors during liver transplantation, 5 min of portal inflow occlusion was found to be insufficient to reduce IR injury [46] while 10 min of portal inflow occlusion was shown to reduce post-operative transaminase levels [33]. Three cycles of 5 min have been sufficient to ameliorate IR injury in small animal models [15], but there is no consensus as to what stimulus is required to adequately precondition humans. In this trial, we performed 3 by 10-min cycles. This longer period of limb ischaemia may explain why RIPC has ameliorated IR injury in this trial compared to others which have used 5-min cycles [25, 26]. It is difficult, however, to draw direct conclusions as the target organ to protect and the limb used (upper vs lower) were different. A further trial comparing 5-min cycles against 10-min cycles is necessary to answer this question.

This clinical study was designed as a pilot feasibility RCT to determine whether patients would be willing to be recruited to a limb preconditioning study with the possibility that they would be randomised to a sham and whether there were risks involved in limb preconditioning using a pneumatic tourniquet. A cohort study using a historical control group would have allowed a comparison between a greater number of patients but would not provide the data on recruitment to help design a future large RCT and would introduce bias due to advances in parenchymal transection techniques and intra-operative blood loss management. Secondary end points which could be used to power a future clinical trial aimed at evaluating the efficacy of RIPC in patients undergoing major liver resections were measured. We have demonstrated that the procedure is acceptable to patients and have found no evidence of complications following RIPC. Surprisingly for a pilot study, we have demonstrated a statistically significant benefit to RIPC in terms of lower post-operative transaminases and improved ICG clearance. These pilot data would justify a prospective clinical trial determining whether RIPC can improve clinical outcomes in major liver surgery.
